# Advanced surface modification techniques for titanium implants: a review of osteogenic and antibacterial strategies

**DOI:** 10.3389/fbioe.2025.1549439

**Published:** 2025-03-19

**Authors:** Handong Zhang, Zidong Wu, Zemin Wang, Xinfeng Yan, Xudong Duan, Huaqiang Sun

**Affiliations:** ^1^ Shandong Key Laboratory of Rheumatic Disease and Translational Medicine, Department of Orthopedic Surgery, The First Affiliated Hospital of Shandong First Medical University & Shandong Provincial Qianfoshan Hospital, Jinan, China; ^2^ Department of Bone and Joint Surgery, The Second Affiliated Hospital of Xi’an Jiaotong University, Xi’an, China

**Keywords:** titanium, biomechanical, surface modification, osteointegration, antibacterial, macrophage polarization

## Abstract

Titanium (Ti) implants are widely used in orthopedic and dental applications due to their excellent mechanical strength, corrosion resistance, and biocompatibility. However, their limited osteointegration and susceptibility to bacterial infections remain major clinical challenges. Recent advancements in surface modification techniques have significantly improved the osteogenic and antibacterial properties of Ti implants. This review summarizes key strategies, including ion doping, hydroxyapatite (HAp) coatings, nanostructured surfaces, and graphene-based modifications. Zinc (Zn)-doped coatings increase osteoblast proliferation by 25%, enhance cell adhesion by 40%, and inhibit *Staphylococcus aureus* by 24%. Magnesium (Mg)-doped Ti surfaces enhance osteoblast differentiation, with 38% increased alkaline phosphatase (ALP) activity and a 4.5-fold increase in cell proliferation. Copper (Cu)-doped coatings achieve 99.45% antibacterial efficacy against *S. aureus* and 98.65% against *Escherichia coli* (*E. coli*). Zn-substituted HAp promotes mineralized nodule formation by 4.5-fold and exhibits 16.25% bacterial inhibition against *E. coli*. Graphene-based coatings stimulate bone marrow stem cells (BMSCs) and provide light-responsive surface potentials for enhanced osteogenesis. Despite these advancements, challenges remain in optimizing ion release kinetics and long-term stability. Future research should focus on multi-functional coatings that integrate osteogenic, antibacterial, and immunomodulatory properties to enhance clinical performance and patient outcomes.

## Highlight

• Innovative Surface Modification Strategies: Integrating metal doping, HAp coatings, and graphene modifications to significantly enhance osseointegration, antibacterial properties, and biocompatibility.
• Synergistic Effects of Multifunctional Coatings: Exploring interactions among metal ions, HAp, and graphene composite coatings to optimize osteogenesis, antibacterial efficacy, and mechanical properties.
• Addressing Clinical Translation Challenges: Focusing on ion release control, long-term stability, and large-scale manufacturing to advance personalized implants and clinical applications.

## 1 Introduction

With the rapid advancement of materials science and medicine, implantable biomaterials have become widely used in orthopedics and dentistry. These materials must exhibit not only excellent biocompatibility but also superior biomechanical properties and long-term stability *in vivo*. The interdisciplinary integration of material science and medicine has driven advancements in implantable biomaterials, particularly through computational techniques that predict and optimize material properties. In addition to these advances, computational methods have also played a pivotal role in optimizing biomaterial performance (Wang et al., 2024). Additionally, innovations in material design have significantly enhanced the biocompatibility and mechanical performance of implants, paving the way for next-generation biomedical applications (Huang et al., 2022a). The surface properties of materials, as the interface in direct contact with biological tissues, play a crucial role in their performance in the body. Pure titanium and its alloys exhibit a diverse array of application domains within the biomedical field, attributed to their exceptional mechanical and chemical attributes, superior corrosion resistance, and exceptional biocompatibility. These qualities render them highly favored in various contexts, notably including osseointegration, bone fixation procedures, and joint replacement surgeries (Basova et al., 2021). Despite their widespread clinical application, titanium (Ti) implants face critical challenges, including inadequate osteogenesis, osteoconduction, and osseointegration (Bandyopadhyay et al., 2023). To address these limitations, extensive research has focused on surface modification techniques, which aim to enhance implant performance by improving biocompatibility, promoting osseointegration, and imparting antibacterial properties.

Titanium implants are primarily secured within the bony matrix through physical bonding, yet they are susceptible to gradual loosening or detachment over extended periods (Zhang et al., 2022). To overcome these challenges, novel composite coatings have been developed with multifunctional attributes. Recent research has explored multifunctional composite coatings that integrate antimicrobial and osteogenic properties. Among these approaches, nanostructured materials have demonstrated the potential to enhance both the antibacterial efficacy and biocompatibility of titanium implants, thus mitigating the risks of post-surgical complications (Wang et al., 2023; Wang et al., 2024). The integration process of these implants with bone tissue is a slow one, typically spanning a duration exceeding 3–6 months. Consequently, the application of titanium implants is constrained by the protracted recovery timeframe resulting from this delayed osseointegration. Moreover, the natural oxide layer present on the titanium surface impedes the direct interaction of early osseointegrating materials with bone tissue, thereby influencing the formation of new bone (Sánchez-Bodón et al., 2021). Thus, the enhancement of implants to facilitate effective osseointegration remains a perpetual concern in the field.

Furthermore, prolonged exposure of titanium implants to human body fluids can elicit adverse biochemical reactions at the implant-bone tissue interface. These reactions can lead to electrochemical corrosion, ultimately shortening the lifespan of the titanium implant (López-Valverde et al., 2022). Surface modification of titanium implants presents a straightforward yet potent approach to mitigate these challenges. By preserving the inherent advantages of titanium implants while introducing novel interfacial attributes, surface modification can augment antimicrobial efficacy, osseointegration, and corrosion resistance.

Recent advancements in surface modification techniques frequently emphasize the incorporation of bioactive trace elements into the implant structure. Trace elements, including calcium, zinc, silicon, magnesium, and strontium (Sr), exhibit the capacity to potentiate osteoblast activity, thereby fostering bone healing and growth (Chouirfa et al., 2018). Additionally, metallic elements such as copper (Cu) and silver (Ag) are ubiquitous in titanium surface modification due to their established antibacterial properties. Moreover, a diverse array of metal elements can stimulate angiogenesis, a critical process for bone repair and regeneration (Zhang et al., 2023). Consequently, these metal elements have been incorporated into titanium surface modification strategies to promote bone repair and osteogenesis.

However, it is often observed that a single elemental incorporation cannot address the multifaceted properties required for optimal implant performance. Therefore, the deposition of two or even three elements onto the implant surface simultaneously has emerged as a focal area of research. The multifaceted approach aims to harness the synergistic benefits of various elements, thereby enhancing the overall performance of titanium implants in terms of osseointegration, corrosion resistance, and antibacterial properties. By exploring and refining these surface modification techniques, the field significantly advances the efficacy and durability of titanium implants in clinical applications (Nazari and Abdelrasoul, 2022).

The conventional calcium phosphate (CaP) coating exhibits commendable biocompatibility and osteogenic conductivity, yet it falls short in terms of bone induction capability and antibacterial efficacy. The unique composition and crystalline lattice of HAp lend themselves to diverse ion substitutions, thereby enhancing its functional properties. Consequently, substituted HAp coatings, through ion substitution, impart supplementary attributes such as bone induction and antibacterial activity. That versatility not only fosters accelerated bone healing during the early implantation phase but also holds promise in the management of various pathological conditions (Arcos and Vallet-Regí, 2020).

Electrical stimulation has emerged as a potent stimulus for augmenting the osteogenic differentiation potential of bone marrow stem cells (BMSCs), thereby accelerating bone regeneration (Jing et al., 2019). Recent advances in photoresponsive implants have leveraged graphene-based scaffolds, which exhibit superior control over cellular activities under optical stimulation. These materials have shown promise in promoting tissue regeneration, particularly in challenging repair scenarios like nerve damage and bone defects (Zhao et al., 2024). When subjected to external stimuli, including electric, magnetic, and optical fields, specific materials exhibit distinct electrical characteristics that generate surface potentials, which can be manipulated by modulating these external fields. Among those fields, light induction stands out due to its superior spatial and temporal control, coupled with enhanced safety profiles compared to electromagnetic or thermal fields, making light-responsive implants a preferred alternative (Wang et al., 2022). However, conventional bone repair implants typically lack photoresponsive properties. Hence, the surface modification of implants using photoresponsive materials, such as graphene and its derivatives (GDs), TiO_2_, and carbon nitride (C_3_N_4_), has captured significant research attention (Wang et al., 2019).

A plethora of studies have delved into various methodologies for modifying implant surfaces through mechanical, chemical, and biological approaches. These investigations have elucidated the optimal mechanical, chemical, and biological parameters conducive to promoting osseointegration. The objectives of this review are to compile and critically analyze studies focusing on surface treatments of titanium and its alloys. Furthermore, it aims to present innovative strategies for augmenting osteogenesis and enhancing osseointegration of implants through surface modification or coating techniques. By synthesizing findings from recent advances in surface modification techniques, including those utilizing computational predictions, multifunctional coatings, and photoresponsive materials, this review aims to bridge the gap between theoretical understanding and clinical application (Wang et al., 2024; Zhao et al., 2024).

The primary novelty of this study lies in the integration of multi-layered surface modification techniques that combine ion doping, nanostructured coatings, and composite coatings to enhance the overall performance of titanium implants. By focusing on synergistic effects from multiple approaches, this study aims to optimize not only osteogenesis and osseointegration but also antibacterial properties and corrosion resistance. Additionally, the incorporation of emerging materials such as graphene and light-responsive materials in surface coatings opens new avenues for promoting bone regeneration and tissue integration. This study proposes a multi-layered surface modification strategy to enhance the mechanical strength, bioactivity, and antimicrobial properties of titanium implants.

## 2 Titanium surface modification

Surface treatments for titanium implants can generally be categorized into two main approaches: (1) surface modifications, which alter the intrinsic properties of the titanium itself, and (2) coatings, which involve depositing an additional layer onto the implant surface. The term *coating* pertains specifically to the application of a novel substance onto the titanium substrate, thereby forming an auxiliary layer on its surface. This process aims to enhance or alter the surface properties of the titanium without significantly modifying its bulk structure. Conversely, surface modification encompasses techniques that involve altering the internal microstructure of the titanium itself, often to improve specific material properties or responses to external stimuli. This distinction is crucial in understanding the various methodologies employed to tailor titanium surfaces for diverse applications, each category offering unique advantages and potential drawbacks in terms of performance, durability, and cost-effectiveness (Chouirfa et al., 2018).

### 2.1 Zinc-doped titanium surface

Zinc stands as an indispensable trace element in human physiology, playing a pivotal role in various cellular processes. Its significance extends to facilitating cell development, catalyzing the synthesis of enzymes and DNA, and participating in the biomineralization process, as evidenced in prior research (Chasapis et al., 2020). Consequently, recent investigations have unveiled that the incorporation of zinc into biomaterial surfaces elicits a positive impact on osteogenic differentiation. Specifically, such modifications augment osteogenesis, improve the biomechanical properties of the implant and enhanced the antibacterial properties of the implant. *In vitro* studies have demonstrated that zinc-doped titanium surfaces significantly enhance osteoblast viability and differentiation compared to non-doped titanium surfaces. Specifically, the incorporation of zinc into the coating resulted in a substantial increase in osteoblast viability, as indicated by higher osteogenic activity and ALP expression, as well as improved cell proliferation and adhesion. The Zn^2+^ and F^−^ co-doped hydroxyapatite (HA) coating exhibited 1.5 times higher osteoblast viability than the pure HAp coating after 7 days of cell culture. Additionally, the Zn-HAp coating (doped with zinc only) reduced the viability of *Staphylococcus aureus* by approximately 24% ± 3% after 24 h, 28% ± 4% after 48 h, and 26% ± 2.5% after 72 h, compared to the pure HAp coating (Bhattacharjee et al., 2022).This highlights the dual benefits of zinc incorporation in both promoting osteogenesis and providing antimicrobial protection (López-Valverde et al., 2022; Wu et al., 2020).

Furthermore, ZnO manifests robust broad-spectrum antibacterial properties, complemented by an array of additional attributes such as antitumor activity and relatively low toxicity. *In vitro*, ZnO coatings demonstrated an antibacterial efficacy of 99.45% against *S. aureus* and 98.65% against *Escherichia coli*, showcasing its excellent broad-spectrum antibacterial properties (Sirelkhatim et al., 2015; Sun et al., 2023). These multifaceted characteristics render ZnO a highly favorable option for the surface modification of titanium substrates. This underscores its potential in enhancing the biocompatibility and functionality of titanium-based implants and devices, thereby expanding the scope of biomedical applications.

Zhang and his colleagues made a significant breakthrough by successfully depositing zinc onto a macroporous coating previously formed on a titanium (Ti) surface through micro-arc oxidation treatment. This deposition was meticulously accomplished through a sequential application of hydrothermal and thermal treatments, ultimately resulting in the development of a layered micro/nanostructured coating termed the micro-arc oxidation-hydrothermal-thermal zinc (MHTZn) coating (Zhang et al., 2019).

The resultant MHTZn coating, comprising a TiO_2_/ZnO composite, constitutes a robust system that harmoniously integrates the wear and corrosion resistance properties inherent in TiO_2_ with the controlled release of Zn^2+^ ions at an optimal rate. This controlled release mechanism is key to maintaining minimal cytotoxicity (Zhang et al., 2018). The Zn^2+^ ions, once released, exhibit a bioregulatory function by interacting with the Runx2 transcription factor. The interaction stimulates osteoblasts, thereby enhancing bone formation through the upregulation of differentiation-related genes (Suhas et al., 2021).

Compared to a TiO_2_ coating alone, the TiO_2_/ZnO coating is more hydrophilic. The TiO_2_/ZnO coating’s ability to adsorb proteins was improved by the material’s increased hydrophilicity, which made it easier to adsorb fibronectin. *In vitro* tests demonstrated that the TiO_2_/ZnO group showed a 25% increase in osteogenesis, including a marked increase in ALP activity, collagen secretion, and upregulation of osteogenic markers osteopontin (OPN) and OCN, compared to the TiO_2_ group (Wu et al., 2023).

In recent years, researchers have found that attracting immune cells, especially macrophages, to the implant site can provide a suitable immune microenvironment for bone regeneration, which plays an important role in the success of implants (Chen et al., 2019). Zinc is essential for both the innate and adaptive immune systems. TiO_2_ nanotubes (TNT) have an excellent osteogenic function and good immune regulation ability (Taleb et al., 2021). Meanwhile, the TNT coating is also an optimal loading and releasing a platform for inorganic elements such as silver and trace elements such as zinc. Therefore, Chen *et al.* fabricated zinc-doped TiO_2_ nanotubes on titanium (Ti) implants to evaluate nanoscale topography and the effect of zinc on the behavior of mouse RAW 264.7 macrophages. They also investigated the effect of zinc-doped TNT surface-regulated macrophages on the behavior and osteogenic differentiation of mouse MC3T3-E1 osteoblasts. In comparison to the inflammation model group, the results showed that the expression of pro-regenerative marker genes and proteins was increased while the expression of pro-inflammatory marker genes and proteins was moderately inhibited in macrophages cultivated on the zinc-doped TNT surface. *In vitro* experiments demonstrated that the zinc-incorporated TNT group significantly outperformed the control group, with enhanced osteogenic gene expression, cell proliferation, and adhesion, as well as increased ALP activity and extracellular mineralization. Among the different Zn-TNT groups, the 15VZn group exhibited the highest ALP activity and osteoblast viability, while the 25VZn group showed the greatest COL1a1 gene expression and mineralization ability (Chen et al., 2020).

When investigating implant materials, it is hoped that one material will have more of the implant’s capabilities. However, the majority of materials currently in broad usage have only one effect, either antibacterial or osteogenic activity (Wu et al., 2022). Nevertheless, the absence of the other effect can still lead to implant failure. Therefore, in order to increase the success rate of implantation procedures, it is necessary to discover biomaterials with strong osteogenic activity and antibacterial capacity (Liu et al., 2024).

**FIGURE 1:**
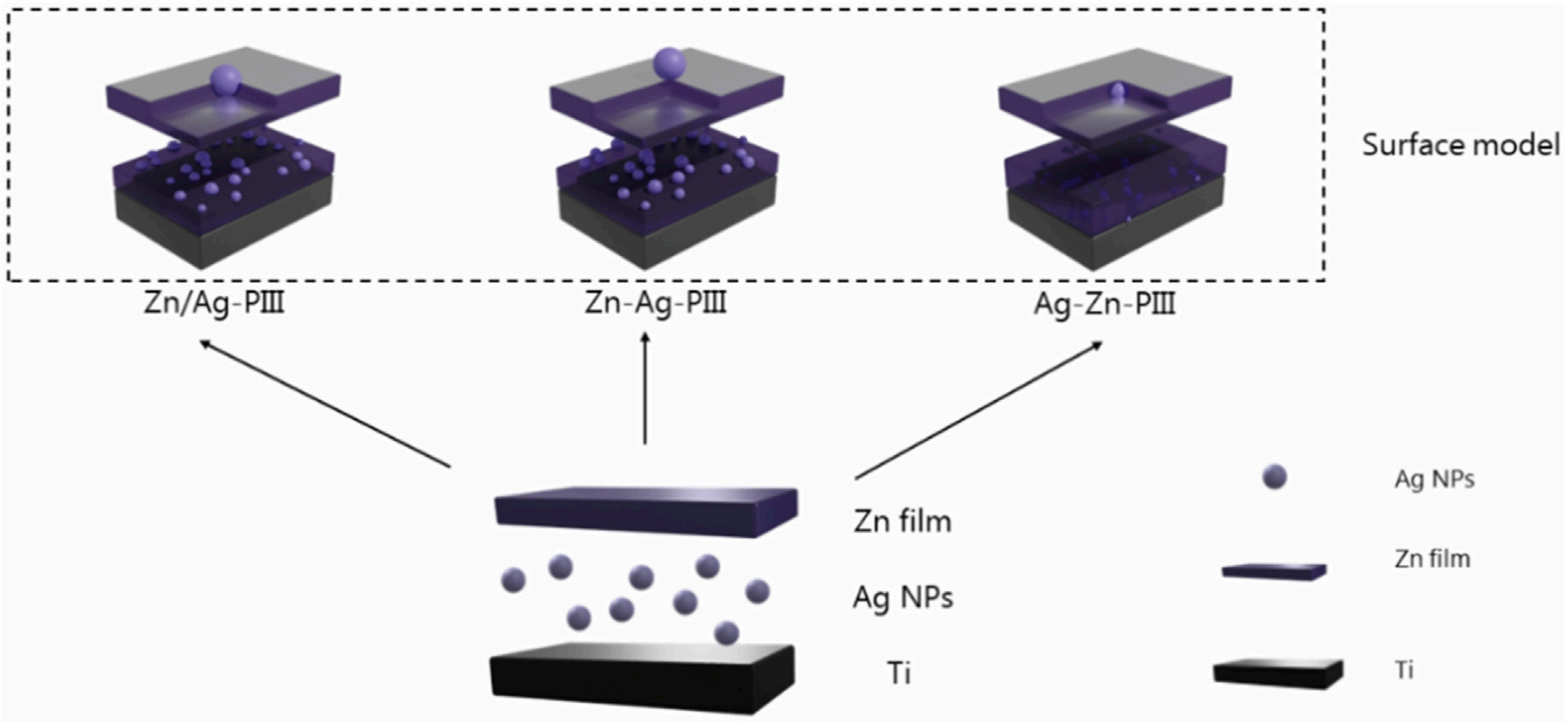
Simulation of surface modelling and corrosion processes of three types of Zn/Ag microelectric couples formed on Ti by Zn/Ag double ion implantation.

Jin and his colleagues innovatively engineered Zn/Ag micro-galvanic couples onto titanium substrates through the advanced technique of plasma immersion ion implantation. Their objective was to establish three distinct surface morphologies of these Zn/Ag micro-galvanic couples, each designed to facilitate a comparative analysis of osseointegration efficacy and antibacterial performance, thereby identifying the optimal structural configuration. That three tailored models of Zn/Ag dual ion implantation on titanium surfaces are schematically illustrated in Figure 1 (Jin et al., 2015).

The *in vitro* evaluations demonstrated that the application of various micro-galvanic couples significantly enhanced osteogenic differentiation and gene expression profiles in rat bone mesenchymal stem cells (rBMSCs). Notably, all three micro-galvanic couple configurations exhibited robust antimicrobial activity (Qu et al., 2023). That research finding underscores the broad-spectrum antibacterial potential of the Zn/Ag combinations employed. In addition, *in vivo* studies revealed significant osseointegration by micro-CT and histological experiments, despite the continued presence of bacteria (Chen et al., 2022).

Investigating the mechanism of micro-galvanic couples revealed that the long-range interaction of Zn^2+^ can have a synergistic effect with the short-range interaction of Ag nanoparticles (Ag NPs), enhancing their osteogenic activity and antibacterial characteristics dramatically. And the pH of the microenvironment between the osteoclast and the bone surface has been demonstrated to have a significant influence on cellular processes, with pH = 4.5 being the optimal pH (Liu et al., 2019). The cathodic hydrogen evolution of micro-couples consumes H^+^ and inhibits bone resorption of osteoclasts. The cathodic hydrogen precipitation reaction of the micro-galvanic couples consumes H^+^, which alters the pH of the microenvironment thereby inhibiting bone resorption by osteoclasts (Wang et al., 2021a). As the corrosion current increases, the consumption of H+ increases, with the highest corrosion rate and best osteointegration observed in the Zn/Ag-PIII model. This model also exhibited the best osteointegration effect *in vivo*, significantly outperforming the Ag-Zn-PIII configuration (Chen et al., 2022).

### 2.2 Magnesium-doped titanium surface

Magnesium (Mg) is an essential mineral and one of the most abundant cations in the human body, with approximately 50% of the total Mg content stored in the skeletal system. Within bone tissue, Mg ions play a crucial role in augmenting calcium (Ca) deposition, a fundamental process in bone formation, as previously reported (Wang et al., 2021b). Extensive research has demonstrated that Mg ions stimulate osteoblastogenesis by modulating osteoblast signal transduction pathways, thereby promoting integrin expression and enhancing cellular adhesion. Consequently, incorporating Mg into orthopedic implants has the potential to accelerate bone tissue repair while improving osseointegration (Shao et al., 2022).

Zhao et al. employed the plasma electrolytic oxidation (PEO) method to incorporate Mg into TiO_2_ micropores, forming a microporous nano-coating (Mg-TiO_2_) on the Ti surface. The PEO technique critically depends on the electrolyte composition, which was optimized using calcium gluconate, magnesium gluconate, ethylenediaminetetraacetic acid (EDTA), and sodium hexametaphosphate. This formulation enabled the controlled release of Ca, P, and Mg ions within the microenvironment, significantly altering its pH and subsequently enhancing osteocyte adhesion. Additionally, Mg ions promoted osteoblast spreading through increased actin expression, while Ca^2+^ enhanced cellular activity, and P ions facilitated cell proliferation. *In vitro* experiments demonstrated a 32% increase in osteoblast proliferation and a 41% enhancement in cell adhesion on Mg-TiO_2_ surfaces compared to pure Ti. Furthermore, ALP activity increased by 38% after 7 days of culture, indicating enhanced osteogenic differentiation (Zhao et al., 2019a).

Post-implantation, macrophages at the implant site differentiate into M1 or M2 phenotypes depending on the physicochemical properties of the material (Yu et al., 2022). Specifically, M1 macrophage-associated cytokines exacerbate bone resorption by increasing osteoclast activity, whereas M2 macrophages contribute to bone regeneration and tissue healing (Lee et al., 2021).Qiao et al. fabricated a magnesium ion-doped TiO_2_ nanotube array (MgN) coating on Ti surfaces via anodic oxidation followed by hydrothermal treatment. Both *in vitro* and *in vivo* analyses revealed a substantial reduction in the expression of pro-inflammatory genes IL-1β, IL-6, and TNF-α by 45%, 38%, and 50%, respectively, in the MgN group. Simultaneously, anti-inflammatory genes IL-1ra and IL-10 exhibited a marked upregulation of 47% and 52%, respectively, compared to the control group (Qiao et al., 2020).These findings strongly suggest that the MgN coating possesses immunomodulatory properties, facilitating macrophage polarization toward the M2 phenotype and thereby creating an osteogenesis-conducive microenvironment.

**FIGURE 2:**
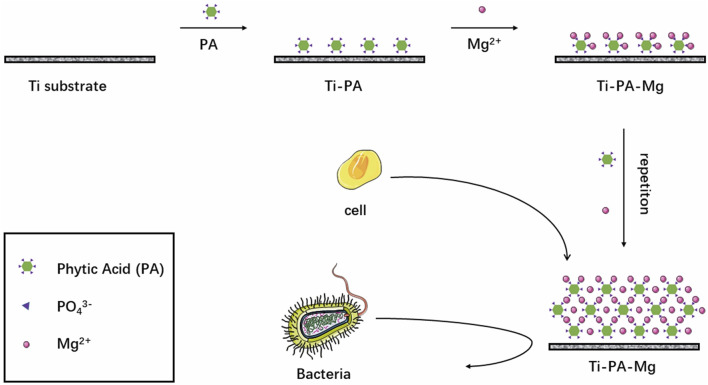
Process of introducing magnesium phytate layer on titanium using phytic acid as crosslinking molecule.

While the incorporation of Mg ions significantly enhances the osteogenic properties of Ti implants, Mg alone lacks inherent antibacterial properties. Phytic acid, a naturally occurring organophosphorus compound, has demonstrated strong chelating abilities with multivalent cations and exhibits notable antimicrobial efficacy. As a result, it has been extensively investigated for surface modification applications (Huang et al., 2021b). To integrate both osteogenic and antimicrobial functions, Liu et al. developed a magnesium phytate (PA-Mg) coating on Ti substrates using phytic acid as a cross-linking agent (Figure 2). The Ti-PA-Mg coating significantly improved surface hydrophilicity, with a water contact angle of 2.77° ± 0.22°, showing a notable enhancement compared to untreated Ti surfaces. Additionally, its protein adsorption capacity reached 0.1402 mg after 24-h immersion in a 1 mg/mL BSA solution (pH 7.4), which was significantly higher than other surfaces. Magnesium ion release tests revealed that Ti-PA-Mg samples released 2.35 ppm of Mg^2+^, whereas Ti-Mg samples released 2.76 ppm. Moreover, the residual magnesium content on the Ti-PA-Mg coating surface was 4.51 At%, significantly higher than 0.27 At% in Ti-Mg. Furthermore, cell proliferation assays demonstrated that the Ti-PA-Mg surface exhibited significantly higher cell proliferation rates on days 1, 3, and 5, with the highest proliferation observed on day 5. Regarding osteogenic activity, the Ti-PA-Mg coating exhibited the strongest ALP activity on days 4 and 14, indicating its superior potential in promoting bone formation and cell growth (Liu et al., 2021).

Furthermore, *in vitro* antibacterial testing demonstrated that the Ti-PA coating achieved a 93.67% inhibition rate against Porphyromonas gingivalis, while Ti-PA-Mg and Ti-Mg coatings showed inhibition rates of 36.17% and 33.17%, respectively. The antibacterial rate of Ti-PA (93.67%) was significantly higher than that of Ti-PA-Mg (36.17%), which may be due to the binding of Mg^2+^ with PA, reducing PA’s chelating ability, thereby weakening its antibacterial effect (Liu et al., 2021).

### 2.3 Cuprum-doped titanium surface

Copper (Cu), a vital trace element essential for human physiological functions, has gained increasing interest in biomedical implants due to its multifunctional role in antimicrobial activity, osteogenesis, and immune regulation (Liang et al., 2022). Considering these concentration-dependent effects, precise control over Cu^2+^ release is crucial to balance its dual role in promoting osteogenesis while avoiding cytotoxicity. Therefore, precise regulation of Cu^2+^ incorporation in biomaterial coatings is essential to ensure optimal antimicrobial efficacy, immune modulation, and osteogenic support (Kartikasari et al., 2022).

Despite its recognized role in osteogenesis, the specific influence of Cu^2+^ on immune-mediated bone regeneration remains underexplored. Since biomaterial surface modifications can significantly influence immune responses, it is crucial to investigate how Cu^2+^-modified surfaces regulate macrophage activity and subsequent bone formation (Dai et al., 2021). Recent studies have demonstrated that Cu^2+^ can modulate macrophage polarization, promoting the transition toward an anti-inflammatory M2 phenotype, which enhances osteogenesis through the secretion of bone morphogenetic protein-2 (BMP-2) and vascular endothelial growth factor-A (VEGF-A) (Laura et al., 2017). This immunomodulatory effect of Cu^2+^ has been particularly highlighted in 3D-printed Ti-6Al-4V-4Cu alloys, where the presence of Cu^2+^ promoted osteogenic gene expression via macrophage-mediated pathways (Wen and Shen, 2021). Given these findings, it is essential to explore biomaterial-based strategies for fine-tuning Cu^2+^ release to optimize its immunomodulatory effects. To this end, Huang et al. developed copper-incorporated micro/nano-morphological coatings on titanium (Ti) substrates to systematically investigate the relationship between Cu^2+^ release and immune modulation. The Cu^2+^ release was tunable, and the optimized coating (MAO-HT2) exhibited enhanced cytocompatibility, further supporting its potential role in immune regulation during bone regeneration (Huang et al., 2019a). Furthermore, the micro/nano-topographic surface architecture of these coatings was found to induce osteogenic differentiation in BMSCs and augment osteoblast maturation, as evidenced by previous studies (Moskalewicz et al., 2022; Xu et al., 2023).

Among various ion-doped coatings, Cu^2+^ has been shown to play a pivotal role in macrophage polarization, yet the precise mechanisms remain unclear. Macrophages, as key regulators of immune responses, exhibit remarkable plasticity and can adopt distinct functional phenotypes depending on environmental cues. The M1 phenotype, typically induced by IFN-γ and LPS, is associated with pro-inflammatory responses and tissue degradation, whereas the M2 phenotype, activated by IL-4 and IL-13, promotes tissue repair and immunosuppression (Murray et al., 2014). Given their crucial role in tissue repair, macrophage polarization significantly influences bone regeneration, with M1 macrophages often associated with tissue degradation and M2 macrophages supporting osteogenesis and tissue repair. Employing these copper-containing micro/nanomorphic coatings as a model system, Huang et al. investigated the modulatory effects of Cu^2+^ on macrophage responses. Their findings revealed a paradoxical dual effect: while the material’s surface characteristics exhibited anti-inflammatory properties, the Cu^2+^ ions released from the coating unexpectedly induced a pro-inflammatory response. This paradoxical effect suggests that Cu^2+^ may exert distinct influences at different biological levels, necessitating further investigation into the underlying mechanisms (Huang et al., 2019b). Therefore, Cu^2+^-modified surfaces could be pivotal in modulating macrophage polarization, which in turn may enhance bone regeneration by promoting a shift toward the M2 phenotype.

Control experiments confirmed that the material’s surface characteristics exhibited an anti-inflammatory effect by regulating integrin (α5, αM, β1, and β2) and Toll-like receptor (TLR-3, TLR-4, Myd88, and Ticam-1/2) signaling pathways. These pathways typically promote tissue repair and bone regeneration by suppressing excessive immune activation. However, in contrast to this anti-inflammatory effect, Cu^2+^ released from the coating activated the copper-transport signaling pathway (CTR1 and ATP7A), leading to macrophage polarization toward the M1 phenotype, which is conventionally associated with inflammation and tissue degradation. Given that M1 macrophages are conventionally associated with inflammatory bone loss, this unexpected Cu^2+^-induced M1 polarization raises questions about its potential effects on osteogenesis (Laura et al., 2017). Interestingly, recent studies suggest that certain inflammatory cues from M1 macrophages, rather than purely inhibiting bone formation, may paradoxically contribute to osteogenic signaling under specific conditions. This has led to growing interest in how Cu^2+^-mediated immune responses interact with osteogenic pathways, particularly through the BMP/Smad axis.

Although M1 macrophages are generally linked to a pro-inflammatory state that suppresses bone formation, recent findings suggest that Cu^2+^-induced inflammation paradoxically enhances osteogenesis through activation of the bone morphogenetic protein (BMP)/Smad pathway. Specifically, the Cu^2+^-induced inflammatory microenvironment upregulates BMP-6, leading to phosphorylation of Smad1/5/8 and subsequent nuclear translocation of RUNX2, a key transcription factor for osteoblast differentiation. To further elucidate this paradox, researchers have examined how Cu^2+^-mediated inflammation regulates key molecular pathways associated with osteogenesis (Pérez and Rius-Pérez, 2022). This observation challenges the classical view that M1 macrophage polarization is detrimental to bone formation, suggesting instead that in certain contexts, inflammation may act as a signaling mechanism to stimulate osteogenic pathways.

This process results in the upregulation of osteogenic markers such as OCN and ALP, thereby promoting bone formation at the bone-implant interface. However, the duration and intensity of inflammation may be critical determinants of whether this response is ultimately beneficial or detrimental to bone healing. One possible explanation for this paradox is that Cu^2+^-induced inflammation differs from chronic pathological inflammation. While prolonged inflammation is known to impair bone regeneration, acute or transient inflammatory responses can serve as critical physiological cues to stimulate osteogenesis. In this case, the Cu^2+^-mediated immune activation might not represent a destructive inflammatory response but rather a short-term osteoimmunomodulatory effect that ultimately supports bone healing (Huang et al., 2019b).

It is worth noting that there is a different perspective here from that presented in Section 2.2 regarding the regulation of the immune system by Mg^2+^. Whereas induction of M2 macrophages was described as helpful for promoting osteogenesis in 2.2, the results here confirm that induction of M1 macrophages is equally beneficial for promoting osteogenesis. It is still difficult to reach a consensus on which phenotype is more favorable to osteogenesis and it is worthwhile to continue to explore that (Strizova et al., 2023).

The co-deposition of two or more metals onto the surface of titanium (Ti) substrates has emerged as a highly favored approach, primarily because a solitary metal ion often falls short in providing the comprehensive array of properties required for effective implant functionality. However, the selection of metals for such composite coatings is not arbitrary. Tantalum (Ta) stands out due to its exceptional corrosion resistance and remarkable biocompatibility, qualities that have led to its widespread utilization as an implant coating to augment osseointegration, as evidenced in previous studies (Balla et al., 2010; Fernandez-Fairen et al., 2010).

Moreover, the nanostructured surface of Ta promotes the adsorption of fibronectin and fosters direct cell-surface interactions, thereby significantly enhancing bone formation when a Ti matrix is coated with nanostructured Ta layers (Sheng et al., 2024). Of particular note, the binary phase diagram reveals a high degree of immiscibility between Cu and Ta. As a result, Cu and Ta can be co-deposited onto the Ti surface without the concern of forming solid solutions or intermetallic compounds (Raeker et al., 2020). This strategic combination not only leverages the individual strengths of Cu and Ta but also avoids the potential pitfalls associated with undesirable phase formations, thus presenting a promising pathway for the development of advanced implant coatings with superior performance characteristics.

**FIGURE 3:**
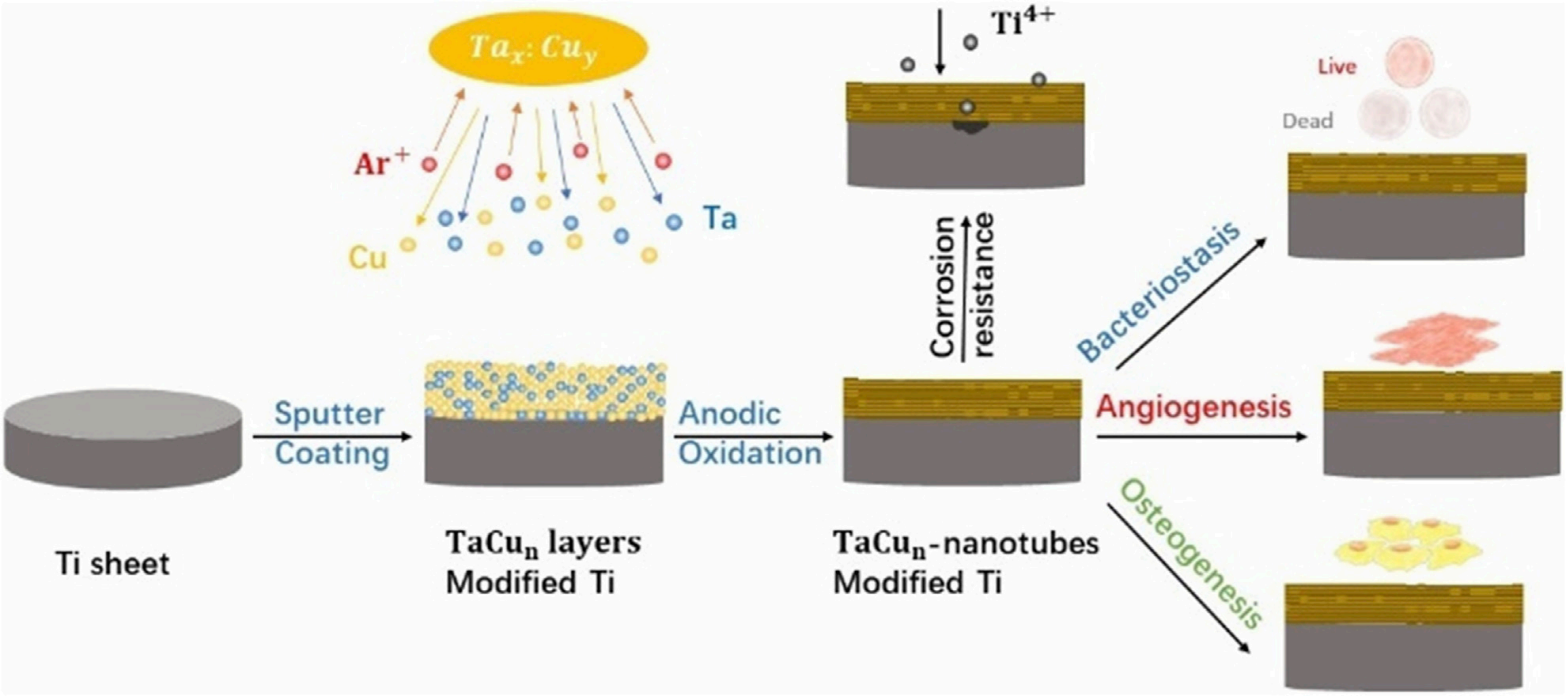
Scheme of the preparation of multifunctional TaCu-nanotubes on Ti substrates with increased corrosion resistance, bacteriostasis, angiogenesis as well as osteogenesis.

Wu *et al.* used magnetron sputtering to deposit varying ratios of Cu and Ta on the surface of Ti substrates to create three coatings. The three coatings were Ta, TaCu1 (Ta: Cu = 4:1 at%) and TaCu2 (Ta: Cu = 1:1 at%), together with a pure titanium substrate to form four control groups. The four material groups were then anodized, yielding four models (Figure 3): TNT, Ta-NT, TaCu1-NT, and TaCu2-NT, which were employed in comparative research. The Cu^2+^ released in the Cu-containing model promotes the migration of human umbilical vein endothelial cells(HUVECs) and enhances the expression of angiogenesis-related genes, thus promoting angiogenesis. In addition, modest Cu^2+^ can improve osteoblast adhesion and spreading. However, osteoblasts are extremely sensitive to Cu concentrations, and high Cu ion concentrations do not promote osteoblast adhesion (Li et al., 2019). The TaCu2-NT model has a relatively high Cu content and a faster Cu^2+^ release early in the implantation process. Therefore, it is not conducive to osteoblast adhesion. Nevertheless, the relatively greater Cu^2+^ release also better enhanced osteogenic gene expression and promoted osteogenesis (Zhang et al., 2019). Furthermore, when the Ta-NT and TNT models were compared, the Ta-containing model exhibited considerable benefits in enhancing the early ALP activity of osteoblasts and promoting mineralization (Wu et al., 2020). In conclusion, the TaCu2-NT model was more effective at promoting osteogenesis and angiogenesis when the four models were compared collectively, particularly comparing the two coatings with various ratios of TaCu1-NT and TaCu2-NT (Wu et al., 2021).

Beyond the functional role of metallic elements in the coating, the implant’s microstructured surface also plays a crucial role in stimulating osteogenesis. To further enhance the osteogenic and antibacterial properties of implants, Zhu et al. developed an advanced surface design methodology. They fabricated precise microgroove patterns on Ti implants (PTi), which provided strong contact guidance to cells, leading to enhanced osteogenic differentiation and bone regeneration. The ALP activity on PTi surfaces was 2 times higher than that of FTi surfaces, while in Ta|TaCu|PTi coatings, ALP expression was further upregulated to 3 times higher than FTi, demonstrating a significant improvement in osteogenic potential. Subsequently, a bilayer nanostructured coating was applied, consisting of an intermediate Ta-5wt%Cu (TaCu) layer for controlled Cu^2+^ release and a top pure Ta layer (Ta | TaCu | PTi) to prevent direct cell exposure to Cu, thus maintaining cytocompatibility. The Ta cap layer significantly reduced Cu^2+^ release, preventing potential cytotoxicity, as evidenced by a controlled and sustained release profile. Additionally, the microgroove design synergized with the bilayer nanostructured coating to significantly increase hydrophilicity, promote cell adhesion and proliferation, and further enhance osteogenic differentiation and bone formation. The osteogenic differentiation markers ALP and RUNX2 showed a 3-fold and 2-fold increase in Ta|PTi surfaces, respectively, confirming the osteoinductive effect. The synergy of these features contributed to an overall 3-fold increase in mineralized nodule formation on Ta|TaCu|PTi coatings, as shown by Alizarin Red S staining (Zhu et al., 2021).

### 2.4 Hydroxyapatite coatings

HAp, with a chemical composition that closely mirrors that of natural bone, possesses exceptional biocompatibility, a crucial attribute for biomedical applications. Moreover, when HAp is synthesized at the nanoscale, it exhibits a remarkable capacity to interact with fibronectin and superconjugate proteins, thereby facilitating robust cell adhesion. The unique property underscores the significance of incorporating a HAp coating layer onto implant surfaces, which has emerged as a widely adopted strategy in the field of implant technology (Li et al., 2024).

Recent studies have shown that deposition conditions significantly influence the performance of HAp coatings. For instance, needle-like nanostructured HAp coatings deposited under pH 6 electrolyte conditions exhibited 92.9% corrosion protection efficiency and demonstrated superior biomineralization, forming three times more new apatite mass *in vitro*. *In vivo* studies further confirmed their excellent biocompatibility, with no significant inflammatory response, highlighting their potential for enhancing bone regeneration (Vladescu et al., 2017).

Lu *et al.* used electrochemical deposition (ED) techniques to create three different nanoscale HA models: nanorod, nanoneedle, and nanoplate HAp coatings (referred to as EDHA-R, EDHA-N, and EDHA-P, respectively), with the goal of discovering the structures with the best osteoinductive and osteogenic activity. When compared to traditional Ti implants, all three EDHA coatings demonstrated higher osteogenic capability. *In vitro*, however, EDHA-P demonstrated greater adsorption of serum proteins than the other two, as well as the cells on EDHA-P also displayed a bigger diffusion area. Additionally, when it came to promoting BMSC proliferation and causing the upregulation of OPN gene expression, EDHA-P outperformed EDHA-R and EDHA-N. *In vivo* tests confirmed these, with the EDHA-P group exhibiting higher osteogenic activity and osteoinductive capacity. Therefore, when putting nanoHA coatings to Ti implants, the EDHA-P model is better (Lu et al., 2020).

Beyond deposition conditions, ion substitution has emerged as another key strategy to enhance HAp coatings. Recent studies have shown that magnesium and zinc substitution significantly improve the bioactivity of HAp coatings by enhancing cell proliferation, biomineralization, and antibacterial properties. Zn-doped HAp coatings (H-Zn) exhibited the highest apatite deposition (14.71 mg) after 21 days in simulated body fluid (SBF), followed by Mg-doped HAp (H-Mg, 14.21 mg) and undoped HAp (13.27 mg). Moreover, cell viability assays demonstrated that H-Zn coatings promoted a 4.50-fold increase in MG63 osteoblast cell viability, outperforming both H-Mg (3.84-fold) and undoped HAp (3.60-fold), indicating enhanced osseointegration potential. In terms of antibacterial efficacy, H-Zn coatings achieved a 16.25% inhibition rate against *Escherichia coli*, while H-Mg coatings exhibited no significant antibacterial effect. These findings highlight the dual functionality of Zn substitution, which not only accelerates bone tissue regeneration but also provides moderate antibacterial protection, making it a promising approach for improving HAp-coated implants (Vranceanu et al., 2024).

The distinctive composition and crystalline lattice of HAp facilitate extensive ion substitutions, thereby imparting it with supplementary functionalities, notably including antibiotic characteristics and bone-inductive capabilities (Arcos and Vallet-Regí, 2020). By integrating HA with silver nanoparticles (AgNPs) and zinc oxide nanoparticles (ZnO NPs), a HAp composite coating can be formulated that concurrently preserves the superior antibacterial and osteoconductive attributes of each constituent material (Ren et al., 2023).This composite design not only leverages the intrinsic properties of HA but also enhances its application value through the synergistic effects of the integrated nanoparticles.

Recent studies have shown that Ag and Sr doped HAp coatings exhibit significant antimicrobial effects in medical applications. The incorporation of silver greatly enhances the antimicrobial performance of the coating, effectively inhibiting the growth of both Gram-positive and Gram-negative bacteria. Additionally, the high surface area of HAp coatings provides an excellent platform for cell attachment and proliferation. Specifically, the nano-HAp structure promotes the differentiation of osteoblasts and the synthesis of bone matrix during the bone formation process. The synergistic effect of silver and strontium not only boosts the antimicrobial activity but also promotes osteogenesis through ion-controlled release, further improving bone integration. Therefore, silver and strontium doped HAp coatings offer dual benefits—antimicrobial and bone regeneration—making them highly promising for medical implant applications (Ungureanu et al., 2023).

**FIGURE 4:**
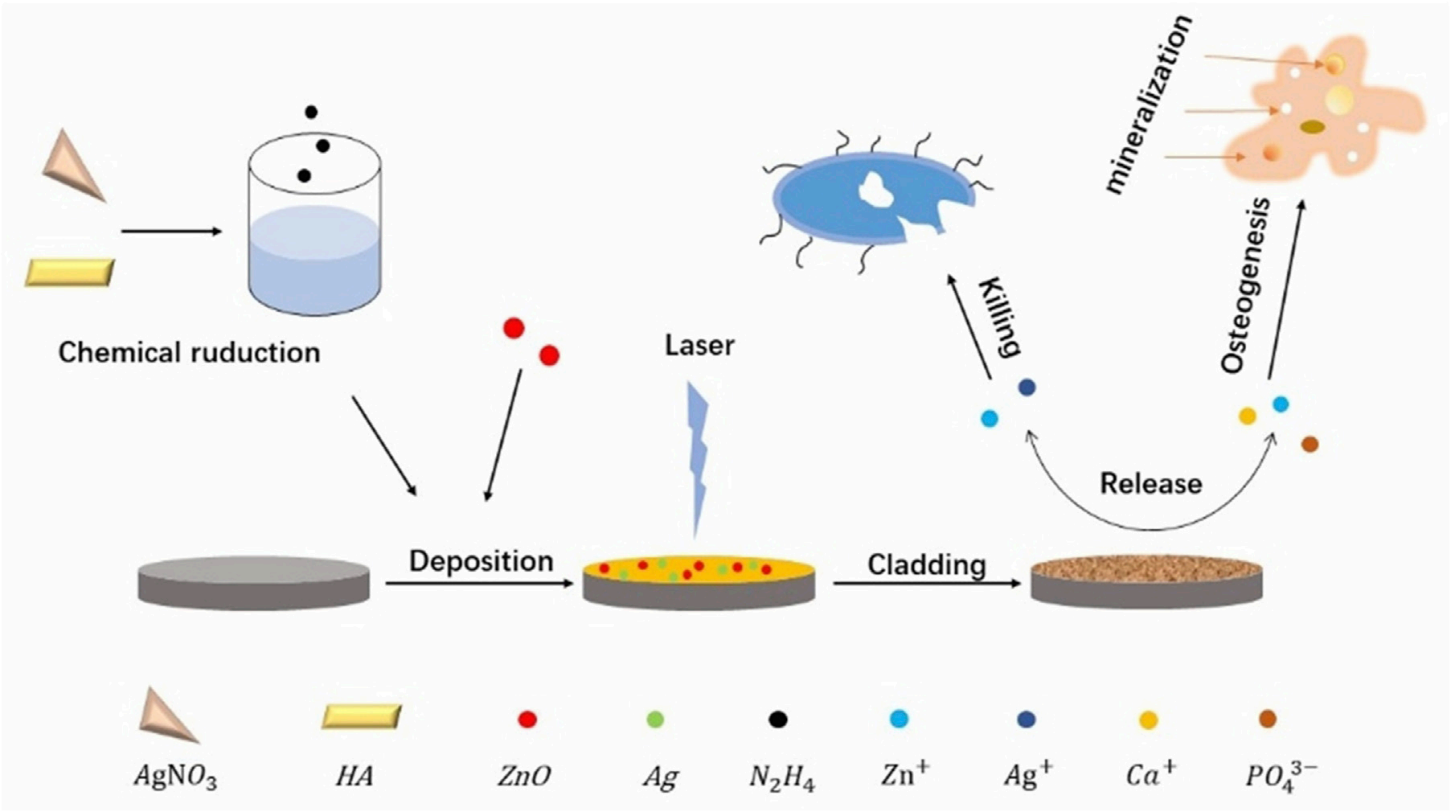
Schematic illustration of the fabrication process of the Ag/ZnO/HA composite coatings on Ti-6Al-4V as well as the bacteria killing and osteogenesis processes.

Jang *et al.* meticulously formulated composite coatings by integrating HAp nanopowders, AgNPs and ZnO NPs in various proportions, opting for the Ti-6Al-4V alloy as the substrate instead of a pristine titanium matrix for their investigative endeavors, as illustrated in Figure 4. The choice of HA, which exhibits a composition akin to that of natural bone, serves a dual purpose. Specifically, it fosters the adhesion and proliferation of osteoblasts and the cells responsible for bone formation. Furthermore, the calcium (Ca) and phosphorus (P) ions liberated by HA stimulate cellular differentiation and mineralization processes, crucial for bone tissue regeneration. Moreover, the incorporation of AgNPs and ZnO NPs within the composite coating enhances its osteogenic potential through the controlled release of Ag^+^ and zinc^2+^. These metallic ions exert a stimulatory effect on osteoblast metabolism, thereby augmenting osteogenesis—a process vital for the synthesis and deposition of bone matrix—and ultimately facilitating robust bone mineralization. The synergistic interplay among these components—HA for its biomimetic properties, and Ag and Zn ions for their metabolic modulation—renders the composite coating exceptionally efficacious in promoting osteogenic activity (Jang et al., 2015).

They employed laser fusion techniques to immobilize the composite coating onto a Ti-6Al-4V substrate to prevent the quick release of Zn^2+^ and Ag^+^ from the composite coating, which can be toxic (Sun et al., 2023). They submerged Ag_x_ZnO_y_HA-Ti6 samples into a simulated bodily fluid to conduct the safety test. According to the experimental findings, after immersion, there is a transient burst of Zn^2+^ release followed by a slowing down gradually of the rate. The Zn^2+^ concentration in the simulated bodily fluid stabilized after 2 weeks and remained below the safe threshold advised by the WHO. In contrast, Ag^+^ was always released slowly and steadily, with no large bursts of release. The cumulative concentration also stayed in the reasonable range, and while larger silver concentrations were more effective in killing germs, they were also more poisonous (Cheng et al., 2023).

### 2.5 Graphene coatings

Graphene and its derivatives have emerged as promising candidates for tissue engineering applications due to their exceptional electrical conductivity, substantial surface area, and intrinsic antibacterial properties coupled with osteogenic induction capabilities (Chen et al., 2024). Specifically, graphene oxide (GO) has demonstrated remarkable efficacy in enhancing and accelerating the osteogenic differentiation of mesenchymal stem cells (MSCs) (Li et al., 2023). Furthermore, GO-modified materials have shown potential in promoting bone formation in their vicinity, highlighting their utility in fostering favorable osteogenic microenvironments (Ding et al., 2023; Hermenean et al., 2017).

The interplay between macrophages and osteoblasts is crucial for assessing the efficacy of bone biomaterials (Tan et al., 2022). Su and his colleagues conducted an insightful investigation to evaluate the impact of GO-coated titanium surfaces on immune cellular responses and the subsequent osteogenic processes. In their study, the titanium (Ti) substrate was initially treated with a robust alkaline solution to engineer a porous surface topography. This surface modification was followed by the introduction of dopamine, which facilitated the formation of a uniform.GO coating on the Ti surface. The experimental outcomes revealed that the GO coating exhibited a promising capacity to augment cellular proliferation. What’s more, the coating demonstrated an enhanced ability to adsorb fibrinogen and vitronectin, proteins abundant in the culture medium and serum, thereby fostering improved cell adhesion and colonization. These findings underscore the GO coating’s potential to create a conducive microenvironment for cellular settlement and growth.

Additionally, the study highlighted a striking effect of the GO coating on the osteogenic differentiation of BMSCs. The results indicated a substantial stimulation in the osteogenic lineage commitment of these cells, suggesting that the GO-coated Ti surfaces could potentially expedite bone formation and integration.

In immunomodulatory terms, they found that immobilized GO coating surfaces induced osteogenic differentiation of BMSCs by activating beneficial immunomodulatory effects of macrophages. In addition, GO coating attenuated the inflammatory response of macrophages, which may favor bone formation around or in GO-modified biomaterials (Su et al., 2020).

**FIGURE 5:**
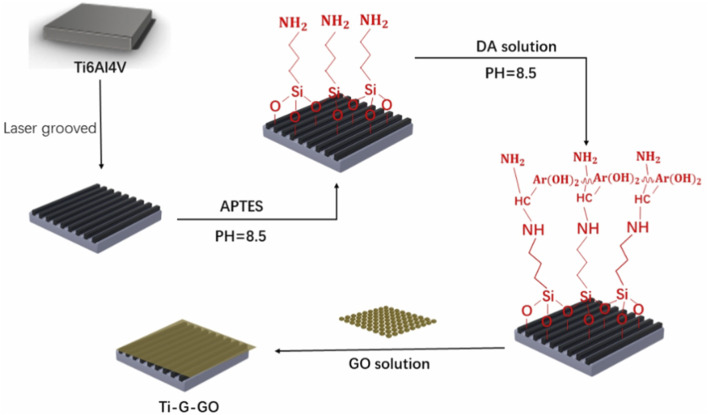
Schematic diagram of the process of making microgrooves in a Ti-6Al-4V plate and fixing the GO coating.

As previously stated, the specific surface structure of the implant, in addition to the coating material, can influence biological processes and hence improve osseointegration (Asensio et al., 2023; Zhou et al., 2023). Laser texturing technique allows for precise carving of the implant surface. Paired with the advantages of surface coating, laser texturing technique can further improve the implant’s osseointegration (Li et al., 2016). Wang et al. created micro-groove patterns on the surface of Ti implants (Ti-G) using laser texturing. Due to the limited adherence of GO to the Ti implant, a transition layer was used before the GO coating was put to the Ti-G to boost adhesion and to avoid coating peeling. Finally, they decided to fix the GO coating on top of the Ti-G (Ti-G-GO) with a double transition layer of 3-aminopropyltriethoxysilane and dopamine (Rahnamaee et al., 2023; Wang et al., 2019) (Figure 5).

The experimental results conclusively demonstrate that the incorporation of textured microgrooves significantly enhances the physical interlocking mechanism between the implant and the adjacent bone tissue. Additionally, the application of a GO coating exhibits superior mechanical attributes, notably enhancing the hardness of the implant surface. The enhancement in surface hardness subsequently facilitates improved bonding with the bone tissue. Furthermore, the GO coating possesses the capability to promote osteogenic differentiation, stimulate cell proliferation, and enhance cellular adhesion, as evidenced in prior literature (Wang et al., 2019).

Many studies have demonstrated that external electrical stimulation can induce osteogenic differentiation of BMSCs. This suggests that generating controlled surface potentials via implants is an effective strategy for regulating osteogenesis (Jing et al., 2019; Wang et al., 2022). With excellent spatial and temporal controllability and safety, photoresponsive materials like TiO_2_, graphene, and its derivatives may generate surface potentials by light stimulation (Bian et al., 2024; Cheng et al., 2017).

Long et al. created an reduced graphene oxide (rGO)/TiO_2_ nanofilm that responds to visible light. They deposited GO/TiO_2_ layers on a Ti substrate. The subsequent reduction of GO to rGO catalyzed by UV light resulted in a heterojunction with the TiO_2_ interface, forming a discharge structure. Under visible light irradiation, a positive surface potential was created on the implant surface. The rGO/TiO_2_ nanofilms were found to be biocompatible, improving BMSC adhesion, and the light-induced surface potential further enhanced BMSC osteogenic differentiation. However, there was little improvement in BMSC proliferation (Long et al., 2021).

## 3 Discussions

Titanium, renowned for its exceptional mechanical strength and biocompatibility, is routinely employed as an implant material. However, its susceptibility to surface oxidation and limited functional efficacy pose significant constraints on the utilization of pure titanium (Ti) implants (Xu et al., 2022). To enhance the success rates of implants and biomechanical properties, it is imperative to integrate additional functionalities, including osteogenesis, angiogenesis, antibacterial properties, and corrosion resistance. Consequently, surface modification of implants has emerged as a focal area of research in recent years, aimed at imparting a diverse array of attributes to address these challenges. One promising strategy for surface modification involves the incorporation of various elements into the coating of Ti implants, enabling them to exhibit multiple functionalities concurrently (Huang et al., 2022b). Several combinations, including silver/zinc, copper/tantalum, zinc/silicon, copper/silicon, strontium/magnesium, and strontium/zinc, have demonstrated remarkable osteogenic and bactericidal effects. The deposition technique plays a critical role in determining the efficiency of these coatings. For instance, plasma electrolytic oxidation (PEO) produces a porous oxide layer that enhances ion release kinetics, making it particularly effective for antibacterial and osteogenic applications (Fattah-Alhosseini et al., 2022). In contrast, magnetron sputtering allows precise control over surface composition and film thickness, but post-deposition thermal treatments are often required to improve adhesion and crystallinity (Stan et al., 2024). Systematic comparisons of these methods are essential to optimize coating performance for clinical applications (He et al., 2020; Zhang et al., 2022; Zhang et al., 2019; Zhao et al., 2019b). Furthermore, the incorporation of biologically active metal ions such as magnesium (Mg), tantalum (Ta), cobalt (Co.), and lithium (Li) into coatings has been shown to augment osseointegration by modulating osteogenic differentiation, extracellular matrix deposition, and osteoclast activity. Among these, Zn-doped surfaces have been reported to enhance ALP activity and upregulate osteogenic markers such as RUNX2 and OCN expression, contributing to improved osteogenesis and bone healing (Dornelas et al., 2024), respectively, while Mg-doped surfaces contribute to collagen synthesis and promote osteoblast adhesion. On the other hand, Cu-doped coatings, though highly effective in reducing bacterial adhesion, require precise ion release control due to their potential inflammatory effects (Nan et al., 2015). Despite these advancements, the selection of optimal element combinations remains challenging, as systematic studies comparing their long-term *in vivo* effects are scarce. Experimental variability, such as differences in ion release kinetics, *in vitro* vs. *in vivo* conditions, and cytotoxic thresholds, further complicates direct comparisons between different metal-doped coatings. Moreover, the complex interplay between metal ions within the physiological environment can lead to adverse chemical interactions, including oxidative stress, local pH fluctuations, and ion competition for cellular uptake, potentially influencing implant stability and biocompatibility. Addressing these challenges requires a comprehensive and systematic approach to assess both individual and synergistic effects of metal ions in real physiological conditions (Peters et al., 2024).

HAp, beyond the extensively utilized metal ions, has emerged as a preeminent coating material for titanium (Ti)-based implants, attributed to its remarkable biocompatibility and bone conduction properties. The intricate composition and crystalline architecture of HAp facilitate numerous ion substitutions, thereby enhancing HAp with supplementary functionalities such as bone induction and antimicrobial activity. However, the choice of deposition technique significantly affects the final coating properties. Plasma-sprayed HAp coatings exhibit high crystallinity and mechanical stability but may suffer from weak adhesion to titanium substrates, requiring surface roughening or additional bonding layers to improve fixation (Li, J. et al., 2023). In contrast, electrodeposited HAp enables better interfacial bonding and tunable ion incorporation, allowing precise control over bioactivity but requiring optimization of deposition parameters to prevent excessive porosity (Mondal et al., 2023). Optimizing these parameters is crucial for achieving long-term implant success. In particular, Zn-HAp coatings have demonstrated superior osteogenic performance, significantly increasing mineralized nodule formation and ALP activity compared to undoped HAp, while Cu-HAp coatings exhibit potent bactericidal effects by disrupting bacterial membranes and promoting ROS generation. The capacity for ion substitution in HAp to immobilize proteins and growth factors opens up novel avenues for the development of hybrid coatings that foster bone healing processes and expedite biomechanical fixation. Moreover, innovative HAp composite coatings have been proposed to address mechanical instability issues, including HAp-graphene hybrids, polymer-reinforced HAp (HAp-PCL, HAp-chitosan), and HAp-bioactive glass composites, which improve fracture toughness, ion release control, and bioactivity. Coupled with advancements in metal scaffold materials, these alternative HAp coatings have broadened the clinical applications of metal implants, transitioning from traditional replacement functionalities to the ambitious goal of bone regeneration (Arcos and Vallet-Regí, 2020). However, a pressing issue is the scarcity of *in vivo* studies on alternative HAp coatings. A pivotal obstacle hindering such research is the unavailability of suitable animal models. To overcome this challenge, future studies should focus on developing more accurate and representative animal models that simulate human bone healing processes. Additionally, combined *in vitro* and *in vivo* approaches can be employed to bridge the gap between experimental conditions and real-world applications. Despite significant advancements in Ti implant coatings, challenges such as long-term biocompatibility, controlled ion release, and large-scale clinical translation remain unresolved. To bridge the gap between *in vitro* studies and real-world applications, large animal models, such as pigs or non-human primates, should be used to validate the long-term performance of modified Ti implants under physiological loading conditions. Additionally, clinical trials should focus on assessing the safety, durability, and efficacy of multifunctional coatings in real-world settings while addressing regulatory challenges, including manufacturing scalability and approval processes. Overcoming these barriers will be crucial for achieving widespread clinical adoption and improving patient outcomes (Bédard et al., 2020). By addressing these issues, we can ensure the more effective clinical translation of advanced HAp coatings, ultimately improving their performance and integration in bone regeneration applications.

Although HAp coatings demonstrate outstanding osteogenesis and biocompatibility, certain limitations remain in terms of antibacterial properties and mechanical performance. For example, while Zn-HAp and Cu-HAp coatings improve osteogenesis and antibacterial capabilities, issues related to mechanical stability and infection resistance still require further optimization. To address these challenges, researchers have turned to graphene and its derivatives, which offer not only superior physicochemical stability but also enhanced bioactivity, making them promising candidates for bone regeneration (Mondal et al., 2023).

Graphene and its derivatives (such as GO and rGO) possess excellent antibacterial properties, mechanical strength, and surface potential regulation, making them highly promising for enhancing osteogenesis and antibacterial efficacy. However, to fully realize the potential of GDs in biomedical applications, it is essential to consider the impact of deposition methods on their final performance. Chemical vapor deposition (CVD) produces high-quality, defect-free graphene coatings with strong mechanical stability, but it requires high-temperature processing and sophisticated equipment, limiting its scalability for large-scale biomedical applications. In contrast, spray coating and dip-coating techniques offer scalable, cost-effective solutions, but they often lead to inhomogeneous coatings with reduced adhesion and mechanical durability. While optimizing deposition techniques is crucial for graphene’s mechanical stability and adhesion, understanding its biological impact on osteogenesis is equally important (Mohammed et al., 2020).

Beyond their physicochemical properties, GDs are particularly intriguing for their ability to actively modulate cellular responses through electrical and biochemical interactions. Their exceptional conductivity enables efficient charge transfer at the cell interface, leading to transmembrane potential (TMP) modulation and activation of intracellular osteogenic pathways. This effect is particularly evident under externally applied electric fields, which enhance GD-mediated modulation of transmembrane potential and subsequent osteogenic signaling. The localized electrical stimulation alters TMP, triggering voltage-gated calcium (Ca^2+^) channel activation. The subsequent Ca^2+^ influx initiates key osteogenic pathways, including BMP/Smad and Wnt/β-catenin, leading to increased Runx2 and ALP expression, thereby promoting osteoblast differentiation and mineralization (Du et al., 2020).

Additionally, GDs indirectly enhance osteogenesis by serving as a platform for the adsorption and stabilization of essential osteogenic stimulants. Their high surface area and functional groups facilitate the electrostatic and chemical adsorption of bone morphogenetic protein-2 (BMP-2) and vascular endothelial growth factor (VEGF), two key regulators of osteoblast differentiation and angiogenesis (Barati et al., 2016). Moreover, GDs interact with immune cells, modulating macrophage polarization and stimulating the secretion of secondary osteogenic factors. Studies show that GO-treated macrophages release oncostatin M (OSM) and VEGF, which further enhance osteogenic differentiation in bone marrow stem cells. Furthermore, GDs adsorb Ca^2+^ and phosphate ions (PO_4_^3-^), facilitating HAp nucleation and accelerating bone matrix deposition (Du et al., 2020).

While these properties highlight the strong potential of GD-based coatings, their successful clinical translation is still hindered by several key challenges. These challenges mirror broader obstacles faced by advanced surface modification technologies, particularly in terms of scalability, regulatory approval, and *in vivo* validation. The scalability of production processes, particularly for complex composite coatings incorporating therapeutic molecules or bioactive agents, remains a critical bottleneck. Moreover, the increasing emphasis on patient-specific implants necessitates highly tailored surface modification approaches, which further complicates the manufacturing pipeline (Xue et al., 2023). Additionally, the lack of long-term *in vivo* validation studies for multifunctional coatings hinders regulatory approval and widespread clinical adoption. Addressing these challenges will be crucial for advancing next-generation titanium implants, ultimately improving patient outcomes and long-term clinical success (Cheng et al., 2021).

## 4 Conclusion

Surface modifications of titanium implants have seen significant advancements, integrating ion doping, bioceramic coatings, and nanomaterial-based modifications to enhance osteogenic potential, antibacterial properties, and biocompatibility. These approaches have successfully improved implant osseointegration and infection resistance, yet key challenges remain in achieving long-term stability, precise control over ion release, and scalable manufacturing.

Among these techniques, metal ion doping and HAp coatings have been extensively studied, demonstrating the potential to enhance osteoblast activity and biointegration. However, issues related to mechanical durability, *in vivo* degradation, and cytotoxicity of certain ions require further optimization. Meanwhile, graphene-based coatings present promising osteogenic and antibacterial effects, but concerns over cost-effectiveness, dispersion stability, and immune response interactions must be addressed before clinical applications.

Despite these advancements, translating these surface modifications into clinical practice remains a formidable challenge. The lack of standardized testing protocols and *in vivo* models for multifunctional coatings hinders regulatory approval. Additionally, patient-specific implant customization demands greater adaptability in coating fabrication techniques, necessitating the integration of advanced manufacturing methods such as 3D printing and biofabrication.

Future research should focus on developing multifunctional coatings that integrate osteoinductive, antibacterial, and immunomodulatory properties, while ensuring controlled degradation and sustained therapeutic efficacy. Scalable, cost-efficient production strategies and long-term *in vivo* validation studies will be essential to facilitate the clinical transition of these promising technologies. By addressing these challenges, next-generation titanium implants will move closer to achieving enhanced clinical performance, improved patient outcomes, and long-term success in biomedical applications.
