# Corrigendum: Advanced surface modification techniques for titanium implants: a review of osteogenic and antibacterial strategies

**DOI:** 10.3389/fbioe.2025.1629360

**Published:** 2025-05-20

**Authors:** Handong Zhang, Zidong Wu, Zemin Wang, Xinfeng Yan, Xudong Duan, Huaqiang Sun

**Affiliations:** ^1^ Shandong Key Laboratory of Rheumatic Disease and Translational Medicine, Department of Orthopedic Surgery, The First Affiliated Hospital of Shandong First Medical University & Shandong Provincial Qianfoshan Hospital, Jinan, China; ^2^ Department of Bone and Joint Surgery, The Second Affiliated Hospital of Xi’an Jiaotong University, Xi’an, China

**Keywords:** titanium, biomechanical, surface modification, osteointegration, antibacterial, macrophage polarization

An image was identified that did not comply with our editorial standards. We have corrected this error and updated the article to reflect this correction.

The Editorial Office apologize for this error and state that this does not change the scientific conclusions of the article in any way. The original article has been updated.

